# What the Surgeon Can Do to Reduce the Risk of Trunnionosis in Hip Arthroplasty: Recommendations from the Literature

**DOI:** 10.3390/ma13081950

**Published:** 2020-04-21

**Authors:** Claude B. Rieker, Peter Wahl

**Affiliations:** 1 Scientific Affairs, Zimmer Biomet EMEA (Europa, Middle East and Africa), Sulzerallee 8, 8404 Winterthur, Switzerland; 2 Division of Orthopaedics and Traumatology, Cantonal Hospital Winterthur, Brauerstrasse 15, 8400 Winterthur, Switzerland; peter.wahl@ksw.ch

**Keywords:** hip arthroplasty, trunnionosis, trunnion failure, fretting corrosion, head–neck junction, mechanically assisted crevice corrosion

## Abstract

Trunnionosis, defined as wear and corrosion at the head–neck taper connection, is a cause of failure in hip arthroplasty. Trunnionosis is linked to a synergistic combination of factors related to the prosthesis, the patient, and the surgeon. This review presents analytical models that allow for the quantification of the impact of these factors, with the aim of providing practical recommendations to help surgeons minimize the occurrence of this failure mode. A tighter fit reduces micromotion and, consequently, fretting of the taper connection. The paramount parameters controlling the fixation force are the coefficient of friction and the impaction force. The influence of the head diameter, as well as of the diameter and angle of the taper, is comparatively small, but varus alignment of the taper and heads with longer necks are unfavourable under physiologic loads. The trunnion should be rinsed, cleaned, and dried carefully, while avoiding any contamination of the bore—the female counterpart within the head—prior to assembly. Biological debris, and even residual water, might critically reduce the fixation of the taper connection between the head and the neck. The impaction force applied to the components should correspond to at least two strong blows with a 500 g hammer, striking the head with an ad hoc impactor aligned with the axis of the taper. These strong blows should correspond to a minimum impaction force of 4000 N.

## 1. Introduction

Total hip arthroplasty (THA) is so successful in restoring mobility and relieving pain in patients with degenerated hip joints [1] that it has been nominated as the operation of the 20th century [2]. Failure, however, remains an issue, with between one-third and nearly half of THA procedures requiring postoperative revision within 30 years [3,4]. One of the possible causes of failure is trunnionosis [5]. Trunnionosis is defined as wear and corrosion at the head–neck taper connection [6]. Hence, it is associated with the modularity of the head–stem construct. Modularity gives the surgeon the flexibility to choose femoral heads of varying materials and diameters, with variable neck lengths, so that the joint replacement can be adjusted according to the patient’s anatomy [7]. It can also reduce the inventory and consecutive storage costs [8,9]. In THA, the use of modular heads began in the early 1970s and has almost completely supplanted monobloc femoral components [6,10].

Epidemiological data on the incidence of clinically relevant trunnionosis are scarce. Up to 4.7% of revisions are reported to be attributable to taper corrosion [11,12,13]. Rates of up to 10.5% are even reported for certain subgroups [12]. While the latter number seems rather high, the occurrence of trunnionosis is certainly underreported, as the taper is seldom analysed. In some revisions, only the femoral head is revised and the stem is retained, making a full analysis impossible. Sometimes, the stem may be revised without disconnecting the head. Additionally, it remains quite difficult to determine the clinical relevance of this issue when other reasons for revision are present concomitantly. While taper corrosion might be observed frequently on retrievals [14,15,16], the clinical relevance might remain difficult to determine [11], particularly because approximately one-third of the adverse local tissue reaction (ALTR) pseudotumours related to taper corrosion identified on magnetic resonance imaging are asymptomatic [17]. A determination of the levels of cobalt and chromium in the synovial fluid might help identify taper corrosion-related issues [18].

The aetiology of trunnionosis is believed to be a synergistic combination of factors related to the prosthesis, the patient, and the surgeon [19,20,21,22,23,24,25]. Trunnionosis involves both fretting corrosion as well as crevice corrosion [26,27]. This process is called mechanically assisted crevice corrosion (MACC) [19]. Taper corrosion can lead to elevated metal ion levels in the synovial fluid of the affected joint as well as in the serum, and may cause ALTR [11,13,18,28,29]. ALTR includes lymphocyte-dominated inflammatory reactions and macrophage infiltrates reacting to particulate corrosion products [11,30]. These can lead to synovitis, local osteolysis, the necrosis of periprosthetic tissues and, finally, component loosening [11,13,31]. Long-term MACC leads to material loss at the taper junction, which can, in rare cases, lead to the frank dissociation of the connection, as well as marked taper deformity [13,20,32,33,34].

This manuscript aims to quantify the impact of these factors according to published analytical models, as well as clinical and in vitro studies, and provide practical recommendations to help surgeons minimize the occurrence of trunnionosis. We conducted a thorough non-systematic review of the literature using two search engines (PubMed and Google Scholar, using the following keywords: trunnionosis, fretting corrosion, taper connection, taper corrosion, taper failure, MACC, modularity, assembly force, disassembly force, micromotion) and cross-referenced related studies to identify the relevant literature.

## 2. Technical Aspects of Taper Connections in Hip Arthroplasty

A taper connection is a means of reliably joining two mechanical components, by tightly fitting a cone into a negative cone-shaped counterpart [35]. The male component is referred to as the trunnion, while the female counterpart is a bore [24,35]. A taper is defined by three parameters: the largest diameter at its base, the smallest diameter at its opening or tip, and its angle [36].

MACC of a Morse taper connection is caused by fretting and crevice corrosion [26,27]. Fretting first disrupts the protective oxide layer on the surfaces of the taper and causes wear. Changes in local chemistry within crevices then lead to the complex interactions of crevice corrosion [26,27]. Although repassivation (i.e., reformation of the protective oxide layers) occurs naturally, fretting alters the repassivation of the exposed metals [24,25,37].

**Figure 1:**
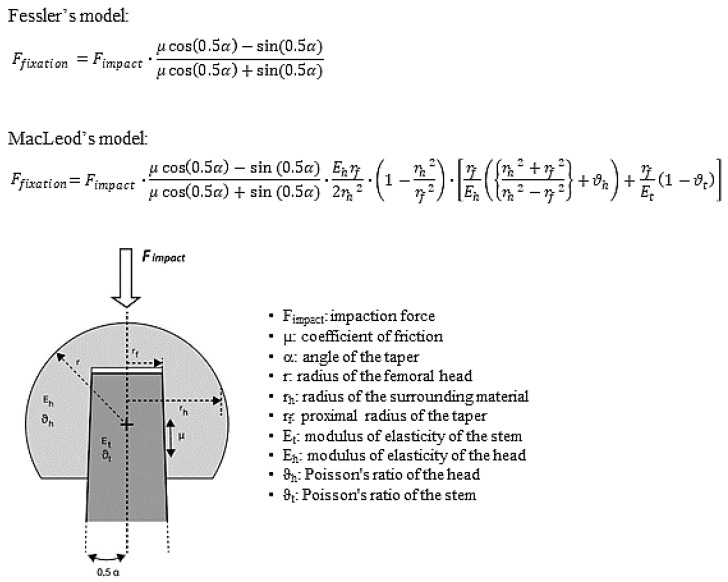
Fessler and MacLeod’s formulas illustrated. MacLeod’s model uses the same fundamental formula as Fessler’s model, as illustrated in the figure, but adds three factors that consider the geometry and the material properties of the system. In both models, the fixation force correlates linearly with the force applied to impact the taper. The effects of the other parameters are illustrated in Figure 2, Figure 3 and Figure 4.

A recently published study indicates that trunnionosis is mainly determined by fretting corrosion, rather than by crevice corrosion [38]. Therefore, minimizing the micromotions at the head–neck taper interface would mitigate the starting conditions of trunnionosis. A strong press-fit fixation of the taper interface will logically lower these micromotions [21,39]. This underlines the importance of a stable fixation between the femoral head and the stem’s trunnion [6,20,21]. Fretting corrosion at the taper interface is linked to micromotions of 5 µm to 12 µm [21,40]. Time in vivo (i.e., exposure to repeated loads) is also linked to the degree of corrosion of tapers [12,26]. Thus, a stable taper connection with lower micromotions under physiological loads will produce a reduced risk of trunnionosis. The force required to remove the head from the taper is a measure of taper stability, denoted as the fixation force or the pull-off force. Given the association between fixation force (i.e., the force necessary to dissociate the taper connection) and micromotion at the taper’s interface [21,39], the fixation force is a surrogate parameter for the rest of this analysis. Fessler et al. [41,42] and MacLeod et al. [43] have both provided analytical models to estimate the fixation force between the neck and the head. These two analytic models are presented in Figure 1.

**Figure 2:**
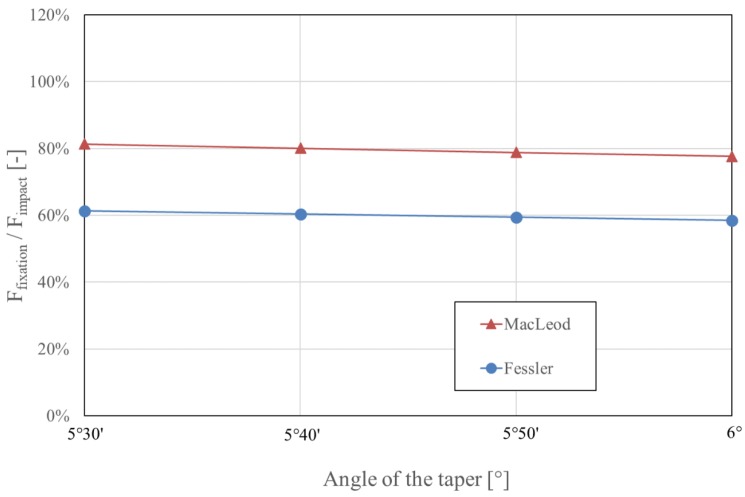
Influence of the angle of the taper on fixation force, as estimated by Fessler and MacLeod’s models. With the impaction force having a linear effect in both models, the fixation force is represented as a proportion of the impaction force. The range of taper angles illustrated covers the range of tapers available commercially in hip arthroplasty. For both models, a coefficient of friction µ of 0.2 was considered. For MacLeod’s estimate, a 32-mm head was considered.

**Figure 3:**
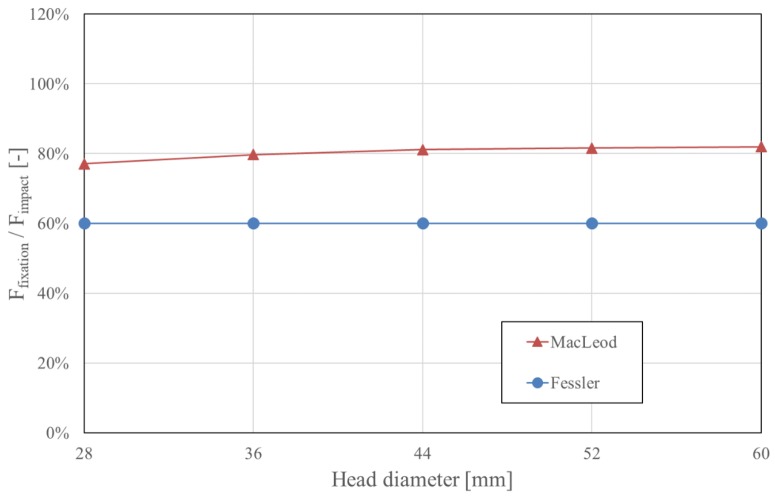
Influence of the diameter of the head on fixation force, as estimated by Fessler and MacLeod’s models. As this parameter is not considered in Fessler’s model, only MacLeod’s model shows a variability depending on this factor. The influence on fixation force, however, remains relatively small and negligible. Notably, MacLeod’s formula provides results with a positive influence for an increasing head size, whereas the measurements describe a decrease of approximately 20% in the fixation force when the head size is increased from 28 to 36 mm. With the impaction force having a linear effect in both models, the fixation force is represented as a proportion of the impaction force.

**Figure 4:**
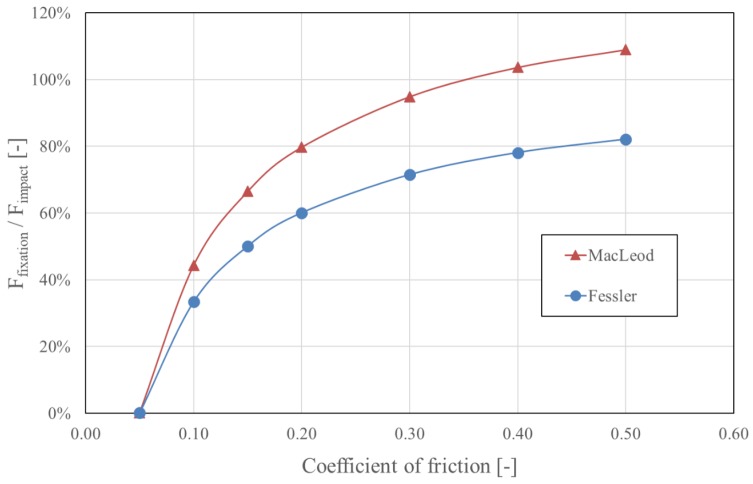
Influence of the coefficient of friction µ on fixation force, as estimated by Fessler and MacLeod’s models. For MacLeod’s model, a head diameter of 32 mm was considered. With the impaction force having a linear effect in both models, the fixation force is represented as a proportion of the impaction force. The effect of the coefficient of friction µ is not linear. With a coefficient of friction < 0.05, stable fixation is not possible. MacLeod’s model not only predicts higher fixation forces than Fessler’s model, it also predicts a fixation force higher than the impaction force starting from a coefficient of friction of 0.372 (for a 32-mm head) and upward, which is physically impossible.

MacLeod’s model is an extension of Fessler’s model and adds three multiplying factors that consider the geometry and the material properties of the system. In both models, the fixation force correlates linearly with the force applied to impact the taper. The fixation force, therefore, has a major effect on the stability of the taper connection [6,44,45,46,47,48,49,50,51,52]. Because the taper angle α is determined by the chosen prosthesis design (typically between 5°30′ and 6° [36]), this parameter has to be considered as fixed. The effect of the taper angle α on the fixation force is negligible, as illustrated in Figure 2. The taper angle α should not be confused with the slope of the taper, which is α/2 [43]. MacLeod’s equation demonstrates that the influence of the diameter of the head (28 mm to 60 mm) is also relatively small, accounting for a maximum variation of 6.4%, as demonstrated in Figure 3. Both models predict that the fixation force is nil for a coefficient of friction of about 0.05, and this fixation force increases as the coefficient of friction increases (Figure 4).

When all other parameters are equal, MacLeod’s model estimates a higher fixation force than Fessler’s model by about a third (Figure 2). However, when considering the technical aspects of the different experimental setups, nearly all fixation force values described in the literature correspond to the values estimated by Fessler’s formula, ranging 40% to 55% of the impaction force [6,21,44,47,48,49,50,51,52]. Surprisingly, the values measured by MacLeod et al. do not correspond to those estimated by their own model [43].

MacLeod’s model is not completely trustworthy for two additional reasons. First, it predicts a higher fixation force than the impaction force, starting from a coefficient of friction of 0.372 (for a 32-mm head) and upward. This would imply the creation of energy within the system, which is clearly impossible. Secondly, while the experimental observations indicated a reduction in the fixation force with an increasing head diameter (with the fixation force of 36 mm heads being 20% less than the fixation force for 28 mm heads [43]), the model predicts the contrary (Figure 3). Considering all these elements, MacLeod’s equation will be omitted for further analysis in this review, and only Fessler’s equation will be considered.

Therefore, the two main parameters controlling the fixation force are the impaction force, which has a linear effect, and the coefficient of friction μ, which should be as high as possible. Under ideal conditions, the value of μ is approximately 0.15 to 0.25 [41,53,54,55]. However, the relationship between the fixation force and the coefficient of friction is not linear. This force becomes nil when the coefficient of friction approaches 0.05 (Figure 4).

Symptomatic trunnionosis appears to be associated with increased head offsets and longer neck lever arms [21,22,56,57]. Micro-grooved tapers, designed to improve stress distributions in ceramic heads, may increase the likelihood of corrosion when combined with metal heads [21,58,59]. However, retrieval studies have not been able to confirm the impact of surface topography, because they have shown comparable fretting corrosion in vivo [21,60]. The variability induced by surface topography is obviously far less important than the other relevant factors in vivo. The association of head diameter with trunnionosis is not always consistent in the literature [12,22,26,57,61,62], but confounding parameters such as time in vivo were not always considered in these studies. Based on some of these publications, large-diameter heads induce force transmission on the taper with a greater lever arm, and this will be mechanically unfavourable when micromotions have to be minimized [43,57,63]. Interestingly, the influence of the head size is not always seen on retrievals [62]. In addition, and perhaps somewhat counterintuitively, the length of the trunnion does not appear to have a significant impact on micromotion or fixation force [47,64]. Increased trunnionosis has also been linked with low flexural rigidity necks (i.e., those with increased elasticity) [65] and small taper angle differences [66]. However, all these parameters are associated with the chosen design of the prosthesis and may not be modifiable intraoperatively. Taper incongruences caused by mixing heads and tapers from different manufacturers might affect the strength of the taper connection and must be avoided, given the variability of the effect associated with cobalt–chromium (CoCr) heads, because taper incongruences may critically reduce the fracture load of ceramic heads [67,68], and because of certification issues. Gross trunnion failures are reportedly above average for tapers made of beta titanium alloy (titanium–molybdenum–zirconium–iron (TMZF)) [34]. This specific beta titanium alloy might have fretting corrosion characteristics that are unfavourable for taper connections.

## 3. Patient-Related Factors for Trunnionosis

Patient-related factors that affect corrosion may include excess body weight [69] and high-impact activities with a resultant increase in demand on the prosthesis [70]. Bergmann et al. have shown that prosthetic loads are several times the patient’s body weight, and the exact load level greatly depends on the patient’s activity [71]. Impact activities greatly increase prosthetic loads; for example, jogging and brisk walking increased the prosthetic load by 3.9 times a patient’s body weight, while stumbling increased the prosthetic load by 11 times a patient’s body weight [71]. These larger loads obviously amplify micromotions at the taper interface. However, the surgeon cannot influence these parameters, as they depend on the patient’s activities in daily life.

The risk of trunnionosis should be considered when planning for THA in patients with larger femoral neck offsets. Varus orientation of the taper and the use of heads with +4 mm necks or longer significantly increase the risk of micromotion and MACC in the taper interface [21]. Given the risk of trunnionosis, implanting the stem in a varus axis to increase the offset might not be mechanically sound. This might well become an issue, particularly with the so-called short stems, which are often recommended for implantation in varus to reconstruct larger femoral offsets [72]. As heads with longer necks might also be detrimental [21], stem designs with larger offsets might have to be favoured. In general, older designs have smaller offsets. When choosing stems with increased offsets, the reduced flexural rigidity of the neck or the taper should be avoided [65,73]. In our opinion, the traditional solution in THA in compensating offset loss with leg-lengthening should be re-evaluated and updated in light of the increasing awareness of trunnionosis.

## 4. Surgeon-Related Factors Determining Taper Fixation

Micromotions in the taper interface, and thus the risk of trunnionosis, are determined by factors under the direct control of the surgeon. These factors are the coefficient of friction and the impaction force, which also appear to be the main determinants of the fixation force, once the prosthetic design has been chosen.

The coefficient of friction is significantly affected by the condition of the trunnion at the time of assembly. This has a major influence on the fixation force, as illustrated in Figure 4. According to several in vitro studies, fluid or fat left on the trunnion at the time of head assembly negatively affect fixation force [10,45,74,75]. By cleaning the trunnion with saline solution and drying it with gauze directly before the assembly of the head, the disassembly forces increase to values observed on pristine control trunnions [10,44,75]. The effect of contamination on the coefficient of friction is independent of the head material (CoCr versus ceramic) [44]. The contamination of the female taper should be avoided carefully while manipulating the head before seating it, as adequate cleaning of the bore would be particularly difficult. A lower coefficient of friction reduces the fixation force and causes higher hoop stresses, increasing the fracture risk for ceramic heads [75]. Drying and cleaning should be done with gauze only, as metallic brushes or pads damage the surface of the taper [76].

The head should never be struck directly with the hammer, but instead with an adequate impactor to avoid damaging the bearing surface of metal heads or fracturing ceramic heads due to point loading. The characteristics of the impactor greatly influence the force transmitted from the hammer blow to the taper connection. Hard plastic tips found on commercially available impactors avoid damage to the head, but they reduce the assembly force by approximately 20% compared to a metallic tip [77]. Older impactors (with questionable stiffness) and impactors with rubber tips should be abandoned [77].

Practical recommendations regarding the impaction force require a discussion of the technical aspects of the studies found in the literature. Quasi-static assembly procedures ensure the precise measurement of the impaction force but do not correspond to the technical solutions available intraoperatively. As the rate of impaction has no relevant effect on the fixation force [49,78], impaction with a hammer might be considered equivalent, even if minor mechanical differences might be identified [55]. While force sensors at the tip of the impactor might approximate the assembly force applied to the taper, the energy dissipated by the impactor must be subtracted from the values measured by the sensing hammers [10,43,50,78,79]. Some studies do not even adequately describe the impaction force used or the fixation force measured [45,74].

The number of hammer blows does not play a significant role, as the impaction force is controlled by the impact with the highest energy [6,45,79]. To ensure the optimal impaction of the taper, two hammer blows are recommended [74]. The first blow could be seen as being the alignment blow and the second blow as being the definitive impaction blow. Many lighter impactions are not useful, as the effect is not cumulative. We recommend a good alignment between the impactor and the neck of the femoral stem. Based on a trigonometric model, a misalignment up to 20° could be tolerated, as 94% of the impacting force would still be maintained in the correct direction. This is confirmed experimentally, with fixation forces not altered significantly by seating loads applied at 20° [52]. Asymmetries of the seating load displacement could, however, be observed at the taper [51,52], and this might explain notable differences in the fixation force observed with off-axis impactions of only 10°, when combined in different planes [80]. Axis deviations of more than 20° are common during THA, at least when using a posterior approach to the hip [81]. Due to the off-axis orientation of physiologic loads, the manual assembly of a taper, relying on later impaction under the patient’s weight, is inadequate [49,70,71].

Impaction with a hammer has its advantages, in that a short impulse causes a lower transmission of energy to the tissues distal to the taper than a slower application of the force [44,55,77]. It might be expected that the impaction of the taper with a hammer is associated with a low risk of femoral fracture, even if high forces are applied. An increased impaction force increases the contact surface, and this has a favourable effect on the stability of the taper connection [39]. However, there is a clinically relevant upper limit to the amount of force applicable. However, to the best of our knowledge, no study has established a maximum recommended force.

Impaction with at least 4000 N is recommended. An adequate impaction force can be reached with a strong blow from a 500 g hammer [46,50]. Surgeons should become familiar and proficient with force-measuring instruments, especially those surgeons with low levels of experience in arthroplasty, to avoid the application of insufficient or off-axis impaction force [46]. The settings of the instrumentation should be checked carefully to ensure that the force measured corresponds to the potential assembly force, and not the force applied with the hammer.

## 5. Discussion

Contemporary hip arthroplasty includes modular heads with variable neck lengths, as this increases the surgical options to tailor the implant to the patient’s individual anatomy and allows for the use of heads made of materials that differ from that of the stem [7]. However, because of the additional interface, modularity may result in additional specific failures. Trunnionosis, defined as fretting and corrosion of the taper connection [6], may result in metal ions and metal particles entering the joint, causing ALTR. Since the first report of a pseudotumour related to taper corrosion was published as early as 1988 [82], corrosion and fretting at the taper junction have become increasingly linked to implant failure [11,12,13,20,22,83]. Surgeons should be aware of all controllable factors to minimize the risk of trunnionosis. This review highlights the important controllable factors that determine the stability of the head–neck taper connection: impaction force and the coefficient of friction at the taper interface.

Fessler’s model quantifies the fixation force of a taper connection [6]. A higher fixation force leads to a tighter fit between components. This will reduce micromotions in the interface between the bore in the head and the stem’s trunnion during physiologic loads, thereby preventing fretting corrosion [10,20,21,40]. The head size is not considered in Fessler’s analytical model of fixation force (Figure 1). Since trunnionosis came into focus as clinically relevant, around ten years ago, multiple factors with a possible impact on the lever arm to the centre of rotation (e.g., large-diameter heads, high mediolateral offset, large neck length, and bearing type) have been discussed as factors that may exacerbate the fretting corrosion process [23,29,58,70]. However, the available data remain inconclusive as to their relevance, and their actual impact remains unclear even to date. Other factors excluded from the analytical model, but under the direct influence of the surgeon, are the impaction technique [6] and the avoidance of mismatch by combining heads and stems with different taper designs (i.e., from different manufacturers) [25,67,68,84].

Modern hip arthroplasty is prone to fretting and corrosion at the taper junction for three reasons. First, procedures are increasingly being performed through small incisions [85], which may impair the proper cleaning and drying of the taper due to reduced exposure. Secondly, driven by the trend to increase the range of motion, there has been a reduction in the diameter of the neck and of the length of the tapers, which reduces flexural rigidity [65,73]. At the same time, head diameters have increased with the aim of reducing the risk of dislocation, along with an increasing range of motion [24,86,87], a change justified by the improved wear characteristics of highly cross-linked polyethylene and ceramic bearings [88,89,90]. Thirdly, these developments have coincided with an increase in obesity rates in most patient populations, with the prevalence of adult obesity exceeding 50% in numerous countries [91]. Taken together, these three developments have created challenges for proper taper fixation and have resulted in corrosion issues.

Finally, there is an increasing understanding of the inter-subject variation in terms of the biologic response to wear particles. Macrophages are activated by wear particles from CoCr alloys [92]. This may lead to the cell-induced corrosion of the taper interface [93]. Improperly seated heads may develop micromotions under physiological loads large enough for macrophages to penetrate the interface and contribute to taper corrosion and failure [52]. This may also be the case with heads engaging the taper proximally, which is the rule for ceramic heads [24]. Differences in alleles are strongly associated with the development of pseudotumours after THA with metal-on-metal bearings [94]. Variations in the genetic signal on the seventh chromosome can influence the probability of developing osteolysis after THA [95].

Trunnionosis develops due to the insufficient fixation of the taper connection. Wear and corrosion alter the surface and the geometry of the trunnion. Despite this, well-fixed stems do not necessarily need to be revised when the trunnion shows severe corrosion. The correct application of a new head does not lead to increased revision rates for corrosion-related issues [11,96,97]. Macroscopic material loss on the trunnion obviously does not allow the proper seating of a new head, and the stem should then be revised. Ceramic heads with titanium alloy inner sleeves may reduce the risk of the recurrence of corrosion issues at the level of the taper compared to CoCr heads [11,97]. However, this remains unconfirmed, considering the difficulties in identifying relevant material issues and the small sample sizes in the studies cited.

In conclusion, trunnionosis is a multifactorial phenomenon related to wear and corrosion in the modular links in hip arthroplasty. A tighter fit decreases micromotion and fretting of the taper interface in the long term and thus reduces the risk of trunnionosis. To minimize micromotions leading to wear and corrosion of the taper connection, surgeons should be aware of the factors directly under their control. Analytical models and empirical investigations reflect the critical significance of carefully cleaning, rinsing, and drying the taper before assembly. The absence of biological residues will yield a higher fixation force between the taper and the femoral head. Fixation strength increases linearly with impaction force. Based on the literature, the adequate impaction of the taper connection can be achieved with at least two strong blows from a 500 g hammer. Surgeons are encouraged to undertake training on force-measuring machines to ensure adequate impaction.
